# Deciphering the interplay of HPV infection, MHC-II expression, and CXCL13^+^ CD4^+^ T cell activation in oropharyngeal cancer: implications for immunotherapy

**DOI:** 10.1007/s00262-024-03789-0

**Published:** 2024-08-06

**Authors:** Shida Yan, Xing Zhang, Qiaohong Lin, Mingyuan Du, Yiqi Li, Shuai He, Jingtao Chen, Xiyuan Li, Jinxin Bei, Shuwei Chen, Ming Song

**Affiliations:** 1 Department of Head and Neck Surgery, State Key Laboratory of Oncology in South China, Collaborative Innovation Center for Cancer Medicine, Sun Yat-sen University Cancer Center, 651 Dongfeng Road East, Guangzhou, 510060 China; 2 State Key Laboratory of Oncology in South China, Guangdong Provincial Clinical Research Center for Cancer, Sun Yat-sen University Cancer Center, Guangzhou, 510060 China

**Keywords:** Oropharyngeal squamous cell carcinoma, HPV infection, Tumor microenvironment, CXCL13^+^ T cells, MHC-II molecule

## Abstract

**Background:**

Human papillomavirus (HPV) infection has become an important etiological driver of oropharyngeal squamous cell carcinoma (OPSCC), leading to unique tumor characteristics. However, the interplay between HPV-associated tumor cells and tumor microenvironment (TME) remains an enigma.

**Methods:**

We performed a single-cell RNA-sequencing (scRNA-seq) on HPV-positive (HPV^+^) and HPV-negative (HPV^‒^) OPSCC tumors, each for three samples, and one normal tonsil tissue. Ex vivo validation assays including immunofluorescence staining, cell line co-culture, and flow cytometry analysis were used to test specific subtypes of HPV^+^ tumor cells and their communications with T cells.

**Results:**

Through a comprehensive single-cell transcriptome analysis, we uncover the distinct transcriptional signatures between HPV^+^ and HPV^‒^ OPSCC. Specifically, HPV^+^ OPSCC tumor cells manifest an enhanced interferon response and elevated expression of the major histocompatibility complex II (MHC-II), potentially bolstering tumor recognition and immune response. Furthermore, we identify a CXCL13^+^CD4^+^ T cell subset that exhibits dual features of both follicular and pro-inflammatory helper T cells. Noteworthily, HPV^+^ OPSCC tumor cells embrace extensive intercellular communications with CXCL13^+^CD4^+^ T cells. Interaction with HPV^+^ OPSCC tumor cells amplifies CXCL13 and IFNγ release in CD4^+^T cells, fostering a pro-inflammatory TME. Additionally, HPV^+^ tumor cells expressing high MHC-II and CXCL13^+^CD4^+^ T cell prevalence are indicative of favorable overall survival rates in OPSCC patients.

**Conclusions:**

Together, our study underscores a synergistic inflammatory immune response orchestrated by highly immunogenic tumor cells and CXCL13^+^CD4^+^ T cells in HPV^+^ OPSCC, offering useful insights into strategy development for patient stratification and effective immunotherapy in OPSCC.

**Supplementary Information:**

The online version contains supplementary material available at 10.1007/s00262-024-03789-0.

## Introduction

Human papillomavirus (HPV) infection is a major risk factor for oropharyngeal squamous cell carcinoma (OPSCC) [1, 2], accounting for over 60% of such cases in the US and nearly 30% in China [3, 4]. HPV-positive (HPV^+^) OPSCC is a distinct disease entity characterized by malignant transformation of the oropharynx due to HPV infection. This unique transformation is accompanied by unique biological behaviors and a more favorable clinical prognosis compared to HPV-negative (HPV^−^) OPSCC, which is caused primarily by conventional carcinogens like alcohol or tobacco exposure [5, 6]. In the era of immunotherapy, although the abundant presence of immune cells in the tumor microenvironment (TME) of HPV^+^ OPSCC is speculated to induce favorable response to immune checkpoint blockade (ICB) therapy [7, 8], the actual response rates of ICB for patients with HPV^+^ OPSCC were heterogeneous and remained relatively low (approximately 20%) [9, 10], highlighting the urgency to delve deeper into the heterogeneous TME of OPSCC and the development of immune response therein.

Previous studies using histological tests and flow cytometry assays have demonstrated the significance of tumor-infiltrating lymphocytes (TILs) in the prognosis of OPSCC, most notably enriched CD3^+^ T and B lymphocytes [11, 12]. The advance of single-cell RNA sequencing (scRNAseq) has profoundly empowered our capability to depict and scrutinize the TME. Intricate composition of immune cells and stromal cells in OPSCC has been unveiled, including distinct signatures of helper CD4^+^ T cells and B cells in HPV^+^ tumor, fibroblasts with specific prognostic value, and comprehensive cellular communications in the TME [13–15]. In response to the viral oncogenes *E6* and *E7*, HPV^+^ OPSCC instigates intratumoral HPV-specific immune responses, showing distinct characteristics and prognostic significance [16, 17]. Furthermore, HPV-infected tumor cells could actively shape the immune landscape, especially through the regulation of effector T cells [17]. However, the mechanisms orchestrating this HPV-specific immune response remain relatively unclear.

Here, through a comprehensive examination of the single-cell transcriptional profiles of both HPV^+^ and HPV^−^ OPSCC samples, we aimed to delineate the heterogeneous landscapes of TME based on different etiological origins. We then focused on the mechanisms underpinning immune infiltration in OPSCC by analyzing the interaction networks between immune and non-immune cells, further corroborating our findings with in vitro functional assays. Additionally, by merging our results with bulk RNA sequencing (RNAseq) results derived from The Cancer Genome Atlas (TCGA) OPSCC samples, we demonstrated their prognostic implications for patients with OPSCC. Together, our in-depth analysis provides valuable insights into the mechanisms underlying the development of immune response and potential therapeutic strategies for OPSCC.

## Materials and methods

### Patient recruitment and sample collection

Six patients who were histologically diagnosed with OPSCCs were enrolled in this study. All patients received surgical resection of their primary tumors after the initial diagnosis and no history of cancer or any anti-tumor therapy before was reported. In addition, a sample of normal tonsil was obtained during the operation of a glossectomy on a patient with tongue cancer after his written consent. The pathological staging was determined based on the 8th edition of American Joint Committee on Cancer (AJCC) staging manual [18]. Fresh tumor samples were obtained during the operation procedure. The HPV infection status was confirmed by HPV DNA detection and genotyping test using a qualitative PCR-based assay. Among them, patients OP01-OP03 were HPV 16 positive while patients OP04–OP06 were found negative. The clinicopathological characteristics of the patients are summarized in Supplementary Table 1. The study was approved by the Institutional Review Boards of Sun Yat-sen University Cancer Center (SYSUCC).

### Preparation of single-cell suspensions

After resection, fresh samples were immediately cut into small pieces with approximately 1 mm [3] in Dulbecco’s Modified Eagle Medium (DMEM; Gibco, USA; Cat. No. 11965092) with 10% fetal bovine serum (FBS; Gibco; Cat. No. 10099141), and then enzymatically digested using Collagenase II (Gibco; Cat. No. 17101015) and IV (Gibco; Cat. No. 17104019) for 30 min on a rotator at 37 °C. Then, the digested mixture was passed through a 40-µm cell-strainer (BD Biosciences, USA; Cat. No. 352340) to obtain dissociated cells and centrifuged at 400 × g for 5 min. After removal of the supernatant, the pelleted cells were resuspended in 0.8% NH_4_Cl red blood cell lysis buffer (Sigma-Aldrich, USA; Cat. No. 254134-5G) and incubated on ice for 10 min. After washing twice with DPBS (Gibco; Cat. No. 14190250), the dissociated cells were resuspended in a sorting buffer consisting of 1X DPBS supplemented with 0.04% BSA (Sigma-Aldrich; Cat. No. 9048468). Viable cells with negative staining of propidium iodide (Thermo Fisher, Cat. No. P1304MP) were collected using fluorescence-activated cell sorting (FACS; BD FACSAria III; BD Biosciences) and at least 300,000 cells in each sample were obtained.

### Single-cell RNA sequencing and raw data processing

Details on library construction, single-cell RNA sequencing, raw data processing, and quality control could be seen in our previous reports [19, 20]. Briefly, approximately 20,000 cells per sample were loaded into a CHROMIUM instrument (10 × Genomics, CA, USA) and mixed with barcoded gel beads. After reverse transcription reaction, cDNA amplification for 14 cycles was conducted on a thermal cycler (C1000; Bio-Rad, USA). The cDNA libraries were constructed, respectively, for single-cell gene expression. Subsequently, libraries were sequenced via Illumina HiSeq XTen instruments (Illumina, USA) with pared-end reads of 150 bp. Raw data from the HiSeq platform were converted into FASTQ files using bcl2fastq (version v2.19.0.316, Illumina). Single-cell 5′-gene expression data were then aligned to the human genome reference sequence (GRCh38) and HPV 16 reference sequence (NCBI NC_001526.4) using Cell Ranger count pipeline (version 4.0.0; 10 × Genomics). Parameters were set as default except for “forcecells” as 13,000. Raw gene expression matrix from Cell Ranger pipelines was converted into Seurat subjects using the Seurat package (version 4.0.2) [21] with the “min.cells” parameter set as 0.1% of all cells and “min.features” parameter set as 500. To remove low-quality cells, we deleted cells with UMIs less than 1000, gene numbers less than 500, or a percentage of mitochondrial genes higher than 0.15. Then, R package DoubletFinder (version 2.0.3; https://github.com/chris-mcginnis-ucsf/DoubletFinder)[22] was applied for each sample to detect and remove potential doublet, with an expected doublet rate of 7.5% and default parameters used otherwise.

### Differential gene expression analysis and cell-type annotation

The remaining cells after quality control were log-normalized and scaled using NormalizeData and ScaleData functions in Seurat. To remove batch effects between each sample, filtered gene expression matrices of all samples were merged using FindIntegrationAnchors and IntegrateData functions in Seurat. Principal component analysis (PCA) and uniform manifold approximation and projection (UMAP) implemented in RunPCA and RunUMAP functions were used for dimensional reduction, and then, the FindClusters function with a resolution of 1.0 was used to identify cell clusters. Then, to define differential expression genes (DEGs) among each cluster, the Wilcoxon test implemented in the FindAllMarkers function was used, while the significant positive expression was considered if it had an average natural logarithm (ln) fold change of at least 0.25 and a Bonferroni-adjusted *P* value lower than 0.05. Subsequently, DEGs were reviewed and used to annotate major cell clusters. Five clusters were identified including T and NK, B, myeloid, stromal, and epithelial cells. To further identify subclusters and annotated specific cell types, the abovementioned procedures (data normalization and scaling, dimensional reduction, cell clustering, and marker identification) were performed within each major cell type.

### Inferring CNVs in epithelial cells

R package inferCNV (version 1.2.1; https://github.com/broadinstitute/inferCNV)[23] was applied to identify malignant cells in epithelial cells, which had somatic large-scale chromosomal copy number alterations, either gains or deletions. The raw gene expression matrix from the Seurat subject was extracted as the expression file based on the software recommendation and the expression matrix of the epithelial cells in the normal tonsil sample was used as a control reference. The CNV landscape of each tumor sample was generated together and separately with default parameters. To concretely illustrate the alteration of each cell from the normal control and to avoid batch effects, we subtracted 1 from the CNV results from the “infercnv.observations.txt” file of each sample and added the absolute values together as the CNV score of each cell. Cells in the EP_C7 subcluster had significantly lower CNV scores than ones from other subcluster. Integrated with the pathway enrichment analysis, the EP_C7 subcluster was identified as the secretory mucosal cell and cells in other groups were denoted as malignant cells.

### Cancer cell state identification

To evaluate the potential functions of cell clusters and identify the cancer cell state of each epithelial subcluster, we calculated the scores of functional modules for specific cell clusters using the AddModuleScore function in Seurat. The corresponding gene sets for cancer cell state identification were previously reported and summarized in Supplementary Table 2 [24].

### Pathway enrichment analysis

To evaluate the enrichment of certain gene sets and pathways of interest in different cell clusters, we performed pathway enrichment analysis using gene ontology (GO) biological processes and kyoto encyclopedia of genes and genomes (KEGG) pathway databases. To explore the heterogeneous features within T cells and epithelial cells, we applied gene set variation analysis (GSVA, version 1.38.2) [25] with default parameters.

### Developmental trajectory inference

During the developmental processes and functional changes of different cell clusters, cells in different states would present a certain transcriptional dynamic repertoire. To infer the developmental trajectory among specific cell clusters, we applied R package Monocle3 (version 1.0.0, https://cole-trapnell-lab.github.io/monocle3/docs/trajectories/) [26] for CD4^+^ T cells with recommended parameters.

### Transcriptional regulator analysis

To infer the gene regulatory networks during the differentiation of CD4_C3_CXCL13 cells, R package SCENIC (version 1.2.2) [27] was applied to identify potential transcription factors (TFs) among CD4^+^ T cells. As recommended, TF searching was restricted to a 10 k distance centered on the transcriptional start site (TSS) or 500 bp upstream of the TSSs. The regulon specificity scores (RSSs) of TFs in CD4_C3_CXCL13 cells were calculated using the “calcRSS” function in SCENIC. The TF with the highest RSS and enriched in the developmental trajectory of CD4_C3_CXCL13 cells was denoted as the key TF of these cells.

### Intercellular communication analysis

CellPhoneDB software (version 2.1.7) [28] contains a repository of curated receptors, ligands, and their interactions and was used to identify potential ligand-receptor interactions across different cell clusters in our cohort. We only considered ligands and receptors with expression in more than 10% of the corresponding cell clusters (–threshold 0.1), and other parameters were set as default. To explore the difference in ligand–receptor interactions between HPV^+^ and HPV^−^ tumors, we also performed the intercellular communication analysis using data from HPV^+^ and HPV^−^ samples, separately. The significant ligand–receptor pairs were filtered with a *P* value of less than 0.05, and we selected the interaction pairs with biological relevance. Visualization of the interaction network was done using Cytoscape (version 3.7.0).

### Deconvolution of bulk RNA-sequencing data

Bulk RNA-sequencing data of OPSCC from The Cancer Genome Atlas (TCGA) database were downloaded and integrated through the UCSC Xena website (https://xena.ucsc.edu/). The HPV infection status was obtained from the published report (Supplementary Table 3) [29]. To verify the cell-type composition in our cohort and investigate the difference in cell-type composition between HPV^+^ and HPV^−^ samples, we utilized two recommended deconvolution tools with high accuracies [30], the MuSiC algorithm (version 0.2.0) [31] and the CIBERSORTx website (https://cibersortx.stanford.edu/) [32], to deconvolute the bulk RNA-sequencing data in the TCGA OPSCC cohort according to their tutorials.

### Immunofluorescence staining assays

Multiplex immunofluorescence (IF) staining assays were conducted to compare the MHC-II expression between HPV^+^ and HPV^−^ samples. Briefly, formalin-fixed paraffin-embedded (FFPE) tissues of OPSCC were obtained from SYSUCC (Supplementary Table 4). The Sections (5-μm thickness) obtained from FFPE samples were dewaxed, rehydrated, and subjected to high-temperature antigen retrieval. Then, the sections were incubated with 3% BSA at room temperature for 30 min and incubated overnight at 4 °C with the following primary antibodies: anti-MHC Class II (10ug/ml; Abcam; Cat. No. ab55152), anti-EpCAM (1:100; Abcam; Cat. No. ab213500) and anti-p16INK4a (1:100; Affinity; Cat. No. AF0228). Subsequently, the sections were incubated with Cy3-conjugated goat anti-rabbit (1:100; Servicebio; Cat. No. GB21303) and FITC-conjugated goat anti-mouse IgG secondary antibodies (1:100; Servicebio; Cat. no. GB22301). Nuclei were counterstained with 4′-6′-diamidino-2-phenylindole (DAPI; 1:20; Sigma-Aldrich; Cat. No. D9542). Images were captured using a confocal laser-scanning microscope (LSM880; Zeiss, Germany).

### Cell culture and lentivirus infection

The two HNSCC cell lines SSC-9 and SAS were purchased from Cell Bank of Type Culture Collection of Chinese Academy of Sciences, and Shanghai Institute of Cell Biology, Chinese Academy of Sciences, which were cultured in DMEM supplemented with 10% FBS. All cell lines were maintained under standard cell culture conditions at 37 °C in a water-saturated atmosphere of 5% CO_2_. Cell lines were regularly tested negative for mycoplasma using an enzymatic assay kit.

The lentivirus of HPV16 E6 and E7, as well as negative control (NC), were purchased from GenePharma (Shanghai, China). The infection of cells was carried out using polybrene (5 ng/ml; Sigma-Aldrich; H9268). After 24-h infection, the infection mix was replaced by a fresh culture medium. The lentivirus vector can also express green fluorescent protein (GFP), providing rapid visual assessment of the viral infection efficiency after 72-h infection. The cells were selected using puromycin (2ug/ml; Selleck; S7417) for stably transfected cells after infection. Real-time quantitative reverse transcription PCR (qRT-PCR) was performed to determine the transcription level of genes using SYBR Premix Ex Taq kit (Takata, Japan; RR420). The following primers were used for qPCR: HPV16 E6: 5′‑CAGTTACTGCGACGTGAGGT‑3′ and 5′‑ACAGCTGGGTTTCTCTACGTG‑3′. For HPV16 E7: 5′‑TTTGCAACCAGAGACAACTGA‑3′ and 5′‑GCCCAT TAACAGGTCTTCCA‑3′. For β-actin: 5′‑AGAGCTACGAGCTGCCTGAC‑3′ and 5′‑AG CACTGTGTTGGCGTAC‑3′.

### Western blotting

Cells were lysed in cell lysis buffer (Beyotime; P0013) with 1 × protease inhibitor cocktail (Beyotime; P1006) for 30 min. After centrifugation, lysate protein concentration was quantified and followed by protein elution in 1 × loading buffer (Fude Biotechnology, #FD006; Tris-HCI [pH8.5], EDTA, DTT, LDS, glycerol, bromophenol blue, pyronin Y.) and boiling for 5 min. Then, samples were analyzed by western blotting. Total proteins in the supernatant fraction were loaded and separated by sodium dodecyl sulfate–polyacrylamide gel (12.5%) electrophoresis (SDS-PAGE), followed by transfer to polyvinylidene difluoride (PVDF) membrane (Merck Millipore, USA). Blocking was done by immersing the membrane with 5% bovine serum albumin (BSA) in tris-buffered saline and Tween 20 (TBST) buffer for 1 h at room temperature, then incubated with the following primary antibodies at 4 °C overnight: MHC-II (abcam; ab55152; 1:3000) and GAPDH (Ray antibody; RM2002; 1:3000), followed by incubation with a secondary monoclonal antibody of mouse origin (Asbio; AS007; 1:3000). The visualization of proteins was achieved by Fdbio-Dura ECL kit (Fdbio science, Hangzhou, China) and Bio-Rad ChemiDoc Touch (Hercules, CA, USA).

### Co-culture assays

For the co-culture assays of HPV^−/+^ OPSCC cells and CD4^+^ T cell, tumor cells and tumoral infiltrating CD4^+^ T cells were isolated from fresh tumor tissues from patients with HPV^+^ OPSCC or HPV^−^ OPSCC (tumor information was summarized in Supplementary Table 3). CD4^+^ T cells were stimulated with anti-CD3 (5 ug/ml, BD Biosciences; 555,329) and anti-CD28 (5 ug/ml, BD Biosciences; 555,725) in RPMI1640 medium with 10% FBS containing recombinant human IL-2 (Miltenyi Biotec, Germany; 130-097-746) for more than 3 days, and then co-cultured with HPV^−/+^ OPSCC cells at a ratio of 5:1, followed by 72 h of incubation.

### Flow cytometry analysis

To estimate the protein expression of intracellular cytokines CXCL13 and IFN-γ, after the addition of Brefeldin A (5 μg/mL; BD Biosciences; 420,601), cells were washed twice using PBS and stained with CD4 (FITC, Biolegend, 317,408) at 4 °C for 30 min. Cells were washed and fixed using the transcription factor buffer set (BD Biosciences; 562,574), and then stained with CXCL13 (PE; R&D; IC801P) and IFN-γ (APC; Biolegend; 502,512) antibody for 45 min at 4 °C. After washed twice, cells were analyzed by a FACS Aria II flow cytometer (BD Biosciences). Data analysis was performed using FlowJo software.

### Statistics analysis

Statistical analyses throughout this study were performed using R, including two-sided paired Student's t-test, two-sided Wilcoxon signed-rank test, two-sided Spearman’s rank correlation test, two-sided log-rank test, one-sided permutation test, and Chi-square test. Only *p* < 0.05 was considered as statistical significance. IF staining, western blotting, and cell co-culture assays were confirmed in at least three biological replicates.

## Results

### Cell composition landscape in OPSCC TME

**Fig. 1:**
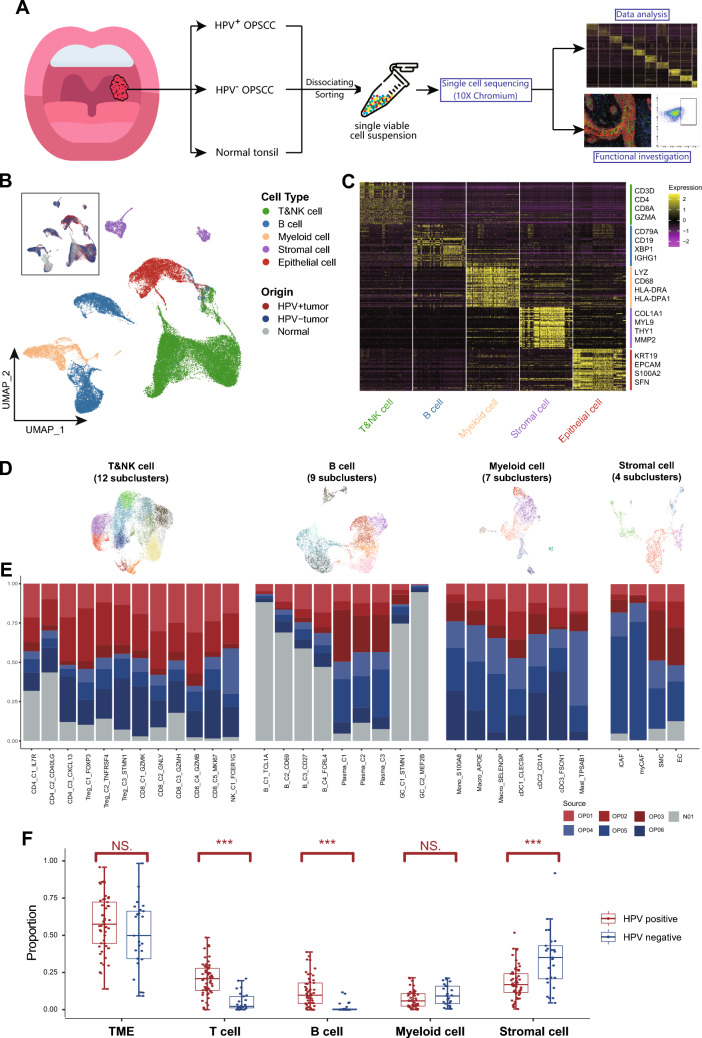
Single-cell landscape of tumor microenvironment of OPSCC. **A** An overview of the study design. **B** UMAP plot showing all 50,161 cells clustered into five major cell types. Each dot represents a cell, which was colored according to its cell type as indicated at the right panel. The inlet plot shows cell distribution colored according to their origins from HPV^+^, HPV^−^ tumor or normal tissue. **C** Heatmap showing the expression levels of top 50 differentially expressed genes (rows) for the five major cell types (columns). The right panel indicating the canonical marker genes defining each major cell type. **D** UMAP plots showing the subclusters of T&NK, B, Myeloid and stromal cells. Each dot represents a cell, which was colored according to its cell type. **E** Bar plots showing the cell proportions from six patients in each subcluster. Among them, OP01-03 were HPV^+^ tumors, OP04-OP06 were HPV^−^ ones, while N01 was normal tonsil tissue. **F** Box plots showing the proportion of each cell type in the samples from TCGA OPSCC cohort using MuSiC algorithm, compared between HPV^+^ and HPV^−^ ones. Each dot represents a patient sample. TME represents the tumor microenvironment including T cells, B cells, myeloid cells, and stromal cells. ***: p < 0.001; NS.: Not significant

We obtained viable cells derived from surgical samples of six newly diagnosed, treat-naïve patients with oropharyngeal squamous carcinoma (HPV^+^ and HPV^−^, n = 3 for each) and one normal tonsil tissue from a consented patient receiving glossectomy (Supplementary Table 1). These cells were subjected to scRNAseq (Fig. 1A). After a strict quality control filtering process, including the removal of cells with low transcripts and inferred doublets, we identified 50,161 cells, with an average of 1600 genes and 5699 unique molecular identifiers (UMIs) expressed in each cell (Supplementary Fig. 1A and B, Supplementary Table 5). By data normalization and unsupervised clustering analysis using Seurat, the cells were categorized into five major cell types according to their expression of canonical markers and the most variable genes, including T and NK (markers: CD3D, CD4, CD8A), B (CD19, IGHG1, XPB1), myeloid (LYZ, CD68, HLA-DRA), stromal (COL1A1, MYL9, THY1), and epithelial cells (KRT19, EPCAM, SFN; Fig. 1B and C, Supplementary Fig. 1C). These major cell types were further classified into distinct subclusters (Fig. 1D). OPSCC samples exhibited multiple subclusters of T, NK, B, myeloid, and stromal cells, with variations in their proportions across patients (Fig. 1D and E). These findings suggest an immune-rich and heterogeneous TME in OPSCC as reported previously [13].

To further elucidate the cell composition of each cell type at a larger scale, we included the TCGA OPSCC cohort (*n* = 78, including 53 HPV-positive tumors and 25 negative ones; Supplementary Table 3) with bulk RNA-seq data available. We utilized the MuSiC algorithm to deconvolve the cell composition of each sample using cell-type specific signatures derived from our study. The overall proportion of TME (including T cells, B cells, myeloid cells, and stromal cells) did not show significant difference between the two OPSCC types (Fig. 1F). However, a significant enrichment of T cells and B cells was evident in HPV^+^ OPSCCs when compared with HPV^‒^ OPSCCs, suggesting an inflammatory TME in the HPV^+^ subtype (Fig. 1F) [33]. In contrast, the HPV^‒^ subtype exhibited a pronounced increase in stromal cells (Fig. 1F), potentially suggesting a mesenchymal and immunosuppressive phenotype in these tumors [34, 35]. To avoid misconceptions by single deconvolution method, we also deconvolute cell compositions using CIBERSORTx, and the results were similar to above findings, with significantly higher compositions of T and B cells in HPV^+^ samples and enrichment of stromal cells in HPV^‒^ ones (Supplementary Fig. 1D).

### Cancer hallmarks of HPV^+^ tumor cells

**Fig. 2:**
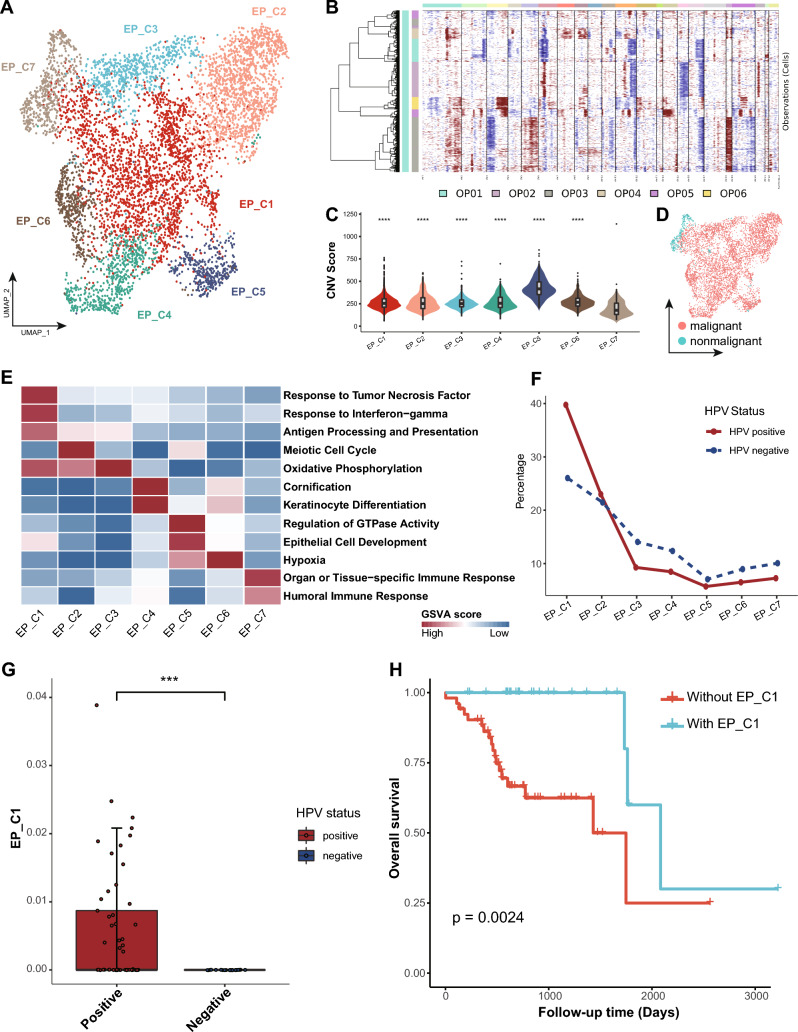
The heterogeneity of epithelial cells between HPV^+^ and HPV^−^ OPSCC. **A** UMAP plot showing 5891 epithelial cells grouped into seven clusters. Each dot represents a cell, colored according to its cell cluster as indicated at the right panel. **B** Heatmap showing the large-scale chromosomal CNVs in epithelial cells from six patient samples. **C** Violin plot showing the CNV score of epithelial cells in each cluster. Comparisons were made using Wilcoxon rank-sum test. ****: *p* < 0.0001. **D** UMAP plot showing the distribution of malignant and non-malignant epithelial cells, colored as indicated. **E** Heatmap showing the selected signaling pathways (rows) with significant enrichment for each subcluster (columns). Filled colors from blue to red in the rectangles represent GSVA scores from low to high. **F** Line chart showing the fraction of HPV^+^ or HPV^−^ cells for each epithelial subcluster. **G** Box plot showing the estimated fraction of EP_C1 in the HPV^+^ and HPV^−^ samples from TCGA OPSCC cohort. Each dot represents a sample, colored according to its HPV status as indicated at the right panel. Comparison between two groups was made using Wilcoxon rank-sum test. ***: *p* < 0.001. **H** Kaplan–Meier overall survival curves of patients from the TCGA OPSCC cohort (N = 78) stratified according to the presence of EP_C1 cells in each sample. Follow-up duration and survival probability are indicated at the xX and Y-axis, respectively. P value was calculated using log-rank test

We identified a total of 5891 epithelial cells in OPSCCs, with 4384 from HPV^+^ OPSCCs and 1507 from HPV^‒^ counterpart, and these cells were grouped into seven clusters (Fig. 2A). InferCNV analysis revealed widespread abnormal chromosome-wide copy number variations (CNVs), instrumental characteristics to differentiating malignant from non-malignant status, within most epithelial cells (EP_C1–C6; see Methods and Supplementary Fig. 2A–F; Fig. 2B–D), suggesting their malignant nature. Moreover, in the HPV^+^ malignant cells, all eight genes in the HPV16 genome were detected. E6 emerged as the most widely expressed, suggesting its oncogenic function in HPV-driven carcinogenesis [36] (Supplementary Fig. 3A). In high-risk HPV types, the primary role of E6 is to drive cell cycle entry and facilitate HPV genome amplification, and its expression is essential for cell proliferation and neoplasia [37].

Gene set variation analysis (GSVA) revealed distinct functions and cell states for each epithelial cluster (Fig. 2E). EP_C1 cells exhibited immune-promoting properties with enhanced response to TNF and IFN-γ, as well as activated antigen processing and presentation (Fig. 2E), suggesting their pivotal role in tumor recognition and immune infiltration [38, 39] (Fig. 2E). Furthermore, this cluster was identified as ‘interferon response’-like cell state [24] (Supplementary Fig. 3B). Interestingly, a comparison between HPV^+^ and HPV^−^ cell distributions across epithelial clusters revealed a significant enrichment of HPV^+^ cells in EP_C1 compared to HPV^−^ cells (Fig. 2F). Moreover, GSEA revealed multiple cancer-related pathways in other malignant epithelial cells (EP_C2–C6), including cell cycle, oxidative phosphorylation, keratinocyte differentiation, hypoxia, and stress, underscoring the transcriptional diversity evident during tumor development (Fig. 2E and Supplementary Fig. 3B) [24]. Additionally, EP_C7 cells were characterized by significant activities in organ or tissue-specific immune response and antimicrobial humoral response, while presented in both HPV^+^ and HPV^−^ OPSCCs. Together with the minimal CNVs detected, this cluster was considered as normal mucosal cells in the tonsil.

In the TCGA OPSCC cohort, EP_C1 cells appeared exclusive to HPV^+^ samples, in contrast to the similar overall composition of epithelial clusters between HPV^+^ and HPV^−^ samples, suggesting a strong correlation between HPV infection and interferon response in HPV^+^ OPSCC (Fig. 2G). Furthermore, survival analysis revealed that patients harboring EP_C1 clusters had significantly better overall survival than those without, suggesting the potential of EP_C1 as a novel prognostic biomarker (Fig. 2H).

### HPV infection and MHC-II expression in tumor cells

**Fig. 3:**
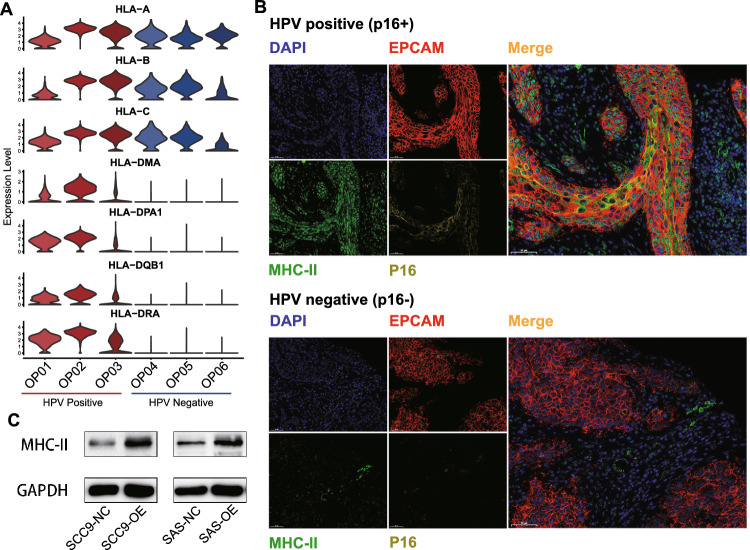
MHC-II expression associated with HPV infection in OPSCC. **A** Violin plots showing the expression levels of MHC-II molecules in the malignant epithelial cells from each sample. The HPV^+^ tumors were colored as red while the HPV^−^ ones were blue. **B** Multiplex IF staining for MHC-II protein in malignant epithelial cells (EPCAM +) in HPV^+^ (top panel) and HPV^−^ OPSCC samples (bottom panel). Images are representative of biological replicates from three patients. **C** Western blotting assay showing the protein expression of MHC-II molecule and GAPDH control in SCC9 and SAS cells with (OE) and without (NC) transfected with HPV-encoded genes E6 and E7

In light of the property of antigen processing and presentation in EP_C1 cells, we conducted differentially expression gene (DEG) analysis between HPV^+^ and HPV^−^ tumor samples to investigate the antigen presentation characteristic in HPV^+^ tumor cells. Strikingly, MHC-II molecule expression was conspicuously higher in HPV^+^ tumor samples. In contrast, MHC-I molecules were broadly expressed across both tumor types (Fig. 3A).

To validate the MHC-II expression in HPV^+^ tumor cells, we conducted multiplex immunofluorescence staining assays on additional OPSCC tumor samples (Supplementary Table 3). A marked MHC-II expression was detected in malignant cells from HPV^+^ tumor samples (with positive p16 staining), compared to its minimal expression in HPV^−^ tumor samples (with negative p16 staining; Fig. 3B). Moreover, to probe the potential causality between HPV infection and MHC-II upregulation, two head-and-neck cancer cell lines, SCC9 and SAS, were transfected with HPV-encoded genes E6 and E7 (Supplementary Fig. 3C and D). Elevated expression of MHC-II molecule (HLA-DPB1) was evident post-transfection (Fig. 3C), underscoring the correlation between HPV infection and MHC-II expression in tumor cells.

### CD4_C3_CXCL13 T cells exhibit dual functions enhancing immune activation

**Fig. 4:**
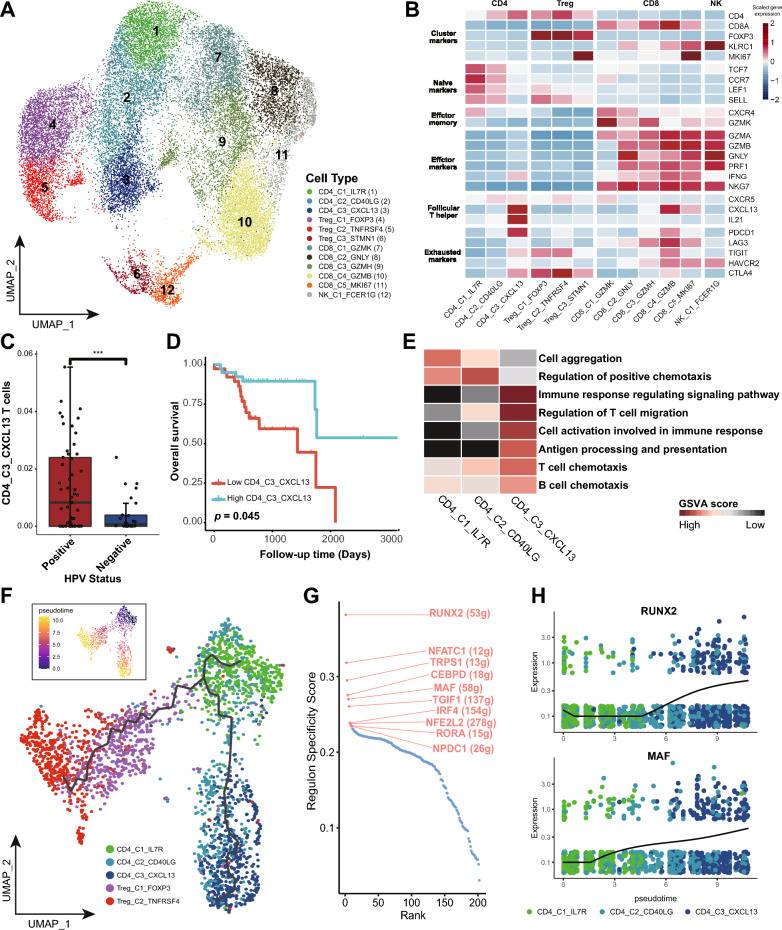
Expression profile and development of CD4_C3_CXCL13 T cells. **A** UMAP plot showing 26,413 T&NK cells grouped into 12 clusters. Each dot represents a cell, colored according to its cell cluster as indicated at the right panel. **B** Heatmap showing the normalized mean expression of canonical marker genes (rows) for each T&NK cluster (columns). **C** Box plot showing the fraction of CD4_C3_CXCL13 T cells in TCGA OPSCC samples grouped into HPV^+^ and HPV^−^ ones, colored according to its HPV status as indicated. **D** Kaplan–Meier overall survival curves of TCGA OPSCC cohort (N = 78) stratified according to their estimated fraction of CD4_C3_CXCL13 T cells. Follow-up duration and survival probability are indicated at the X- and Y-axis, respectively. P value was calculated using log-rank test. **E** Heatmap showing the selected signaling pathways (rows) with significant enrichment for each subcluster of conventional CD4^+^T cells (columns). Filled colors from black to red in the rectangles represent GSVA scores from low to high. **F** Pseudotime development trajectories of CD4^+^T cells. Each dot represents a cell in the trajectory projection, colored according to CD4^+^T cell clusters. The inlet plot shows cells colored according to predicted pseudotime scores from dark purple to yellow, representing cell states from early stage to terminal stage. **G** Scatter plot showing the specificity scores of regulons of CD4_C3_CXCL13 cells. with top 10 highlighted. **H** Scatter plots showing the expression levels of RUNX2 and MAF (Y-axis) along the pseudotime trajectory (X-axis). Each dot represents a cell, colored according to its cell cluster as indicated at the below panel

We identified 26,413 T and NK cells, which were further grouped into 12 subclusters based on classical markers and expression of functional gene modules including three conventional CD4^+^T clusters, three Treg clusters, five CD8^+^T clusters, and one cluster of NK cells (Fig. 4A and B). Given the critical role of MHC-II in presenting antigens to CD4^+^ T cells, we mainly focused on the features and differentiation of CD4^+^ T cells.

Notably, CD4_C3_CXCL13 exhibited high expression of *CXCL13*, *IL21,* and *IFNG*, suggesting their dual roles as both follicular helper *T* (*T*_fh_) cells and T helper 1 (*T*_*h*1_) cells. An elevated expression of *PDCD1* and *CTLA4* further indicated an enhanced immune-regulator function (Fig. 4B). CD4_C3_CXCL13 T cells were significantly overrepresented in HPV^+^ samples from the TCGA OPSCC cohort (*p* < 0.001, Fig. 4C). Survival analysis revealed that an increased proportion of CD4_C3_CXCL13 significantly correlated with favorable OS for OPSCC in the TCGA cohort, consistent with previous studies (*P* = 0.045, Fig. 4D) [13]. Pathway enrichment analysis further unveiled the active role of CD4_C3_CXCL13 T cells in immune response modulation, antigen processing, presentation, and chemotaxis of T and B cells (Fig. 4E). Together, these findings underscore the critical role of CD4_C3_CXCL13 T cells in orchestrating a robust immune-active TME in HPV^+^ OPSCC.

To elucidate the development and differentiation of CD4_C3_CXCL13, we conducted pseudotime analysis for CD4^+^ T cells using the Monocle3 algorithm [24] (Fig. 4F). The analysis revealed a beginning of the differentiation trajectory at CD4_C1_IL7R, followed by two bifurcated development paths toward Treg cells and conventional CD4^+^T cells. Notably, CD4_C3_CXCL13 cells were primarily located at the differentiated stage within the branch encompassing conventional CD4^+^T cells. Single-cell regulatory network inference and clustering (SCENIC) analysis was conducted to identify subcluster-specific TFs that drive the CD4^+^T cells differentiation trajectory, revealing RUNX2 to be the regulon with the highest specificity score in CD4_C3_CXCL13 (Fig. 4G). Furthermore, RUNX2 expression surged along with the differentiation of CD4_C3_CXCL13 from CD4_C1_IL7R (Fig. 4H). These findings are consistent with the notion that Runx family proteins are essential to modulate the development and function of T helper cells [40]. Additionally, MAF is another top-ranking 10 regulons steering CD4_C3_CXCL13 differentiation and is highly expressed in this cell cluster, consistent with its reported role in driving the T_FH_ cell response (Fig. 4G and H) [41].

### Intercellular interactions between HPV^+^ tumor cells and CD4^+^ T cells promote inflammatory signature

**Fig. 5:**
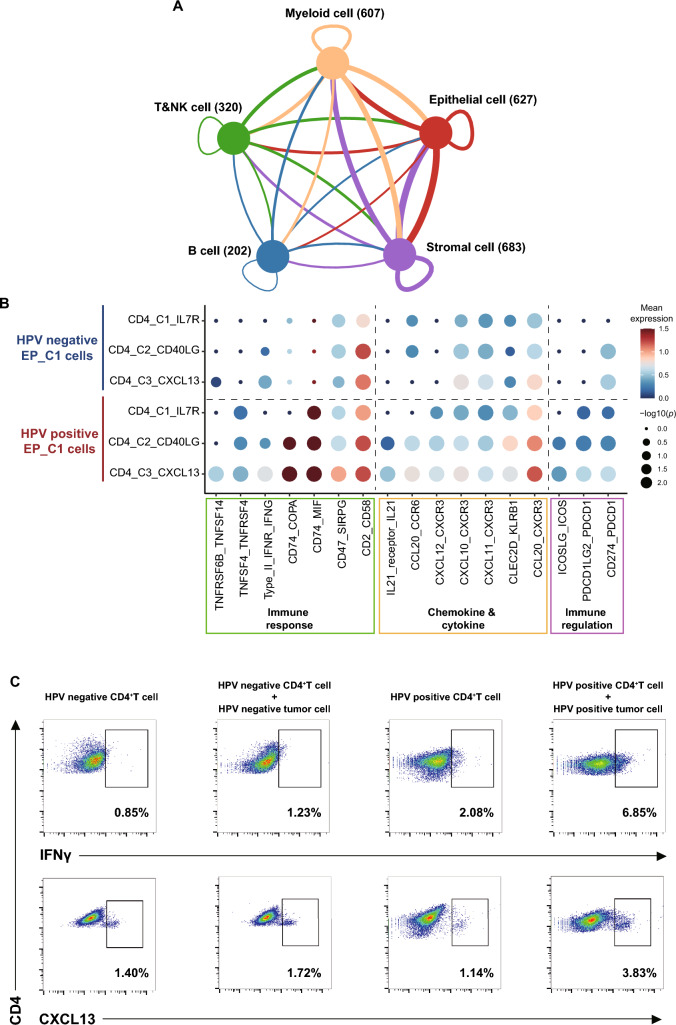
Cell–cell communications between tumor cells and CD4^+^T cells. **A** Ligand–receptor interactions between each major cell type. The thickness of each line represents the interaction intensity estimated between the corresponding two cell types. The number after each cell type indicated the total number of ligand–receptor pairs between the corresponding cell type and other cell types. **B** Dot plots showed selected ligand–receptor interactions between EP_C1 cells and conventional CD4^+^T cells. The ligand–receptor interactions and cell–cell interactions are indicated at columns and rows, respectively. The means of the average expression levels of two interacting molecules are indicated by color heatmap (right panel), with blue to red representing low to high expression. The log_10_(*P*-values) are indicated by circle size using one-sided permutation test. Different color boxes at the bottom represent different function modules of receptor–ligand interactions. **C** Representative flow cytometry analyses for the protein expression of IFNγ and CXCL13 in CD4^+^T cells co-cultured with or without tumor cells from HPV^−^ or HPV^+^ OPSCC tumors, as indicated at the top. The top right boxes show the proportions of CD4^+^IFNγ^+^T cells (upper) and the CD4^+^CXCL13^+^T cells (below), respectively. Results are representative of three biological replicates

Given the specific expression of MHC-II in HPV^+^ tumor cells and its critical link with CD4^+^ T cells, we utilized CellPhoneDB algorithm to explore cellular communications through ligand-receptor interactions between tumor cells and CD4 + T cells (Fig. 5A; Supplementary Fig. 4A and B). Interestingly, we observed much more immunologic interactions between CD4^+^T cells with HPV^+^ tumor cells than with HPV^‒^ tumor cells, including ligand-receptor pairs associated with immune response (CD74-COPA, CD74-MIF, Type II IFNR-IFNγ), chemotactic signals like chemokines and cytokines (IL21-IL21 receptor, CCL20-CXCR3), and those pivotal for immune regulation (CD274-PDCD1, PDCD1LG2-PDCD1; Fig. 5B). Among them, CD4_C3_CXCL13 T cells emerged as the most extensive communicative with HPV^+^ tumor cells, especially through CD74-COPA, CD74-MIF and CD2-CD58, which play a critical role in MHC-II antigen presentation and T cell activation [42, 43]. Additionally, the ligand–receptor interactions between CD4_C3_CXCL13 and both CD8^+^ T and B cells suggest that CD4_C3_CXCL13 could promote immune activation and enhance the T/B cell immunity (Supplementary Fig. 4C and D) [44].

To verify the incited inflammatory response driven by tumor–T cell interactions, we performed functional co-culture assays for CD4^+^T cells and OPSCC tumor cells. Under the ambiance of HPV^+^ tumor cells, CD4^+^T cells manifested an upregulated expression of CXCL13 and IFNγ, indicating the enhanced differentiation toward CXCL13^+^ CD4^+^ T cells and T_H1_ phenotype (Fig. 5C). Contrastingly, when HPV^−^ tumor cells interacted with CD4^+^T cells, such a specialized subcluster remained elusive (Fig. 5C). These findings corroborate the notion that tumor cells, particularly those with elevated MHC-II expression, are essential to induce CD4^+^T cell activation and promote immune response [45].

## Discussion

The recognized favorable prognosis of HPV^+^ OPSCC over the HPV^‒^ counterpart remained an enigma, as it could not be fully explained by the HPV-driven carcinogenesis and related genomic alterations [2]. Through our comprehensive single-cell transcriptomic analysis, we dissected the heterogeneous landscapes of TMEs in HPV^+^, HPV^‒^ OPSCC, and healthy tonsil tissues. Our analysis unveiled an immune-rich TME in OPSCC and a significant enrichment of T cells and B cells, suggesting an inflammatory TME in the HPV^+^ subtype. Notably, we discovered that HPV^+^ OPSCC contains a specific subcluster of tumor cells exhibiting elevated MHC-II expression and antigen-presenting property, which could be further induced by HPV infection. Moreover, HPV^+^ tumor cells expressing MHC-II molecules could directly interact with CD4^+^T cells and promote their differentiation and activation, fostering an inflammatory TME in HPV^+^ OPSCC. Therefore, our study suggests a distinctive TME in HPV-associated OPSCC, which is pivotal to spearhead an HPV-specific anti-tumor immune response contributing to favorable treatment outcomes [11].

Our study uniquely identifies the elevated expression of MHC-II molecules in HPV^+^ tumor cells. While high MHC-II expression in traditional antigen-presenting cells (APCs) has been demonstrated in HPV^+^ OPSCC [46], our study further demonstrates that introducing HPV genes upregulates MHC-II expression in specific cancer cell lines, underscoring HPV infection as a primary driver for enhanced immunogenicity. These findings suggest that the contrast in immune infiltration between HPV^+^ and HPV^‒^ OPSCC might originate from the difference in immunogenicity. Despite a general consensus that tumors tend to downregulate MHC-I expression to evade immune surveillance, the role of tumor cell-specific MHC-II (tsMHC-II) observed in various tumors remains controversial [47, 48]. Our study underlines a strong association between tumor cells with high MHC-II expression and the activation of the IFNγ-mediated pathway, which has been known for both MHC-II production and its hallmark as a T cell-inflamed signature [38, 49]. Additionally, our analysis unveils a robust link between high tsMHC-II expression and positive regulation of T cell proliferation and activation. After stimulated by IFNγ, tumor cells would express transcriptional regulator class II transactivator (CIITA), which induces the expression of MHC-II, through the JAK/STAT signaling pathway [38]. Together, these insights point to a regulatory interplay between IFNγ and MHC-II in HPV^+^ tumor cells, which potentially promotes the anti-tumor T cell immunity. Besides, researchers had found tumor cell-expressed MHC-II as a response predictor to anti-PD-1/PD-L1 therapy [39, 47]. This reinforces the perspective that MHC-II expression in tumor cells might be indicative of a favorable prognosis and a better response to immune checkpoint inhibitors.

Apart from the established cytotoxic capacity of CD8^+^T cells, CD4^+^ T cells emerge as a multifaceted player in anti-tumor immunity [50]. Our cellular network analysis via CellPhoneDB highlights a robust cell–cell interaction between HPV^+^ tumor cells and CD4^+^T cells. Furthermore, HPV^+^ tumor cells could directly induce CXCL13 expression in CD4^+^T cells, differentiating CXCL13^+^CD4^+^T cells, as evident in our co-culture assays. Our study spotlights CD4_C3_CXCL13 cell cluster, characterized by high CXCL13 and PDCD1 expression, as a subset of mature and activated CD4^+^ T cells. These cells closely resemble the dual-functional T cells reported earlier with attributes of both follicular helper (T_FH_) T cells and T helper 1 (T_H1_) cells [51]. Studies showed that the clonal expansion of CXCL13^+^CD4^+^T cells correlated with immunotherapy response and also promoted CD8^+^T cells differentiation [52]. Additionally, in the ambiance of HPV^+^ tumor cells, CD4^+^T cells exhibit enhanced IFNγ secretion, which is critical to orchestrating adaptive immune response against viral and tumoral antigens. Collectively, our findings suggest that HPV^+^ tumor cells could interact with CD4^+^T cells, likely through MHC-II recognition independent of traditional APCs [53], facilitating anti-tumor immunity. Previous reports had found that MHC-II expressed by tumor cells could directly present endogenous antigens to CD4^+^T cells and might through endocytosis [53]. Moreover, after activated by HPV^+^ tumor cells, CD4_C3_CXCL13 T cells were significantly enriched in HPV^+^ OPSCC and have been associated with a favorable prognosis, underscoring their potential as a prognostic biomarker [13].

Although our findings supported the pro-inflammatory role of IFNγ signaling in tumor biology, IFNγ also has pleiotropic effects on ICB. By an “adaptive resistance” mechanism, IFNγ might upregulate expression of PD-L1 and drive T cell dysfunction [54]. As in our study, we also observed co-expression of PD-1 and IFNγ in the CD4_C3_CXCL13 T cell, suggesting its dual functions on pro-inflammation and immune regulation. Besides, IFN signaling in cancer cells was also found to promote cancer cell survival and decrease infiltration of T and NK cells, causing cancer cell intrinsic and extrinsic resistance to ICB [55]. Therefore, it is necessary to deeply investigate the comprehensive role of IFNγ signaling in the TME of OPSCC and resolve the issue of unresponsiveness and resistance to the ICB treatment.

Previous bulk sequencing and single-cell analyses had revealed multiple signatures of TMEs in HPV^+^ OPSCC that might be implicated in prognosis and treatment strategy. For example, several studies had developed immune classification or inflammation score based on TMEs, which might facilitate the clinical decision making [14, 56]. Besides, certain immune cell clusters had been found to affect prognosis of HPV^+^ OPSCC, such as intratumoral CD103^+^ cells and CD4^+^ T follicular helper cells, and the latter ones were consistent with CXCL13^+^CD4^+^T cells in our study [13, 57]. However, the single-cell features of malignant cells were less delineated. A partial-EMT signature was found in HPV^–^ HNSCC as an independent predictor for node metastasis, but HPV^+^ OPSCC was not considered [58]. In our study, similar with previous studies, we revealed an immune-enriched TMEs and specific CXCL13^+^CD4^+^T cells that were associated with prognosis in HPV^+^ OPSCC. Moreover, we focused on malignant epithelial cells and their interaction with immune cells of HPV^+^ OPSCC. Similar to our findings, such tumor cells with MHC-II expression have been reported in other malignancies and suggested prognosis and therapeutic applications [47, 48]. However, further investigation is needed to show the mechanism of tumor cells expressing MHC-II and their interaction with immune cells in HPV^+^ OPSCC.

Together, our investigation revealed a reciprocal relationship between HPV^+^ tumor cells and T cell immunity. As a part of the viral immune response, T cells would provoke extensive expression of cytokines including IFNγ, which might sequentially promote the tsMHC-II expression in HPV^+^ tumor cells [6, 38]. Reciprocally, HPV^+^ tumor cells could directly interact with CD4^+^T cells and activate CD4_C3_CXCL13 T cell differentiation, which also had elevated secretion of IFNγ. Such interactions might further facilitate an immune-infiltrated TME and indicate a better response to immunotherapy. In fact, infiltration of CXCL13^+^T cells and high expression of tsMHC-II were suggested to be potential predictors of ICB responses [39, 59]. However, one significant limitation on this study is that there is no suitable database about OPSCC using the ICB therapy available for evaluating their predictive values in OPSCC, and prospective clinical trials could investigate these feasibilities. Moreover, we recognize that the limited sample size in our single-cell analysis might induce biases in the TME characterization and downplay minor cell subsets in OPSCC. Future efforts require validations on a larger scale, and in-depth experiments are essential to uncover underlying mechanisms and potential therapeutic applications for OPSCC.

## Supplementary Information

Below is the link to the electronic supplementary material.

Supplementary file1 (DOCX 14 kb)

Supplementary file2 (PDF 10086 kb)

Supplementary file3 (PDF 10198 kb)

Supplementary file4 (PDF 9439 kb)

Supplementary file5 (PDF 534 kb)

Supplementary file6 (XLSX 9 kb)

Supplementary file7 (XLSX 27 kb)

Supplementary file8 (XLSX 20 kb)

Supplementary file9 (XLSX 9 kb)

Supplementary file10 (XLSX 9 kb)

## Data Availability

The original contributions presented in the study are included in the article/supplementary material. The raw sequence data reported in this paper have been deposited in the Genome Sequence Archive (Genomics, Proteomics and Bioinformatics 2021) in National Genomics Data Center (Nucleic Acids Res 2022), China National Center for Bioinformation/Beijing Institute of Genomics, Chinese Academy of Sciences (GSA-Human: HRA007637) that are publicly accessible at https://ngdc.cncb.ac.cn/gsa-human.
