# Supplementary material for: Deciphering the interplay of HPV infection, MHC-II expression, and CXCL13+ CD4+ T cell activation in oropharyngeal cancer: implications for immunotherapy
Source: Cancer Immunol Immunother. 2024 Aug 6;73(10):206. doi: 10.1007/s00262-024-03789-0 (PMC11303625; doi:10.1007/s00262-024-03789-0)
Supplement: Supplementary file 1 — Supplementary file1 (DOCX 14 kb) [file 262_2024_3789_MOESM1_ESM.docx]

**Supplementary figure 1.** **A & B.** Box plots showing the number of UMIs (A) and genes (B) for each sample. **C.** UMAP plots showing the distribution of each major cell type. Each dot represents a cell, and the depth of color from light grey to deep purple represents low to high expression of canonical marker genes signature genes. D. Box plots showing the proportion of each cell type in the samples from TCGA OPSCC cohort using the CIBERSORTx website, compared between HPV+ and HPV- ones. Each dot represents a patient sample. TME represents the tumor microenvironment including T cells, B cells, myeloid cells, and stromal cells. ***: p<0.001; NS.: Not significant.

**Supplementary figure 2. A-F.** Heatmaps showing the large-scale chromosomal CNVs in epithelial cells from each sample (OP01-OP06).

**Supplementary figure 3.** **A.** UMAP plots showing the normalized expression of HPV-encoded genes. Each dot represents a cell, and the depth of color from light grey to deep purple represents low to high expression. **B.** Violin plots showing the calculated module scores for interferon, cell cycle, oxidative phosphorylation, squamous differentiation, hypoxia, and stress signaling pathways of each epithelial cluster. **C & D.** Bar plots showing the qPCR results revealing relative expression levels of E6 (C) and E7 (D) in SCC9 and SAS cells, with or without transfected with E6 and E7.

**Supplementary figure 4. A & B.** Ligand-receptor interactions between each major cell type in HPV^+^ (A) and HPV^-^ tumors (B), respectively. The thickness of each line represents the interaction intensity estimated between the corresponding two cell types. The number after each cell type indicated the total number of ligand-receptor pairs between the corresponding cell type and other cell types. **C & D.** Dot plots showed selected ligand-receptor interactions of CD4_C3_CXCL13 T cells with CD8^+^T cells (C) and B cells (D), respectively. The means of the average expression levels of two interacting molecules are indicated by color heatmap (right panel), with blue to red representing low to high expression. The log_10_(P-values) were indicated by circle size in one-sided permutation test.
