# Supplementary figures and images for: Deciphering the interplay of HPV infection, MHC-II expression, and CXCL13+ CD4+ T cell activation in oropharyngeal cancer: implications for immunotherapy
Source: Cancer Immunol Immunother. 2024 Aug 6;73(10):206. doi: 10.1007/s00262-024-03789-0 (PMC11303625; doi:10.1007/s00262-024-03789-0)

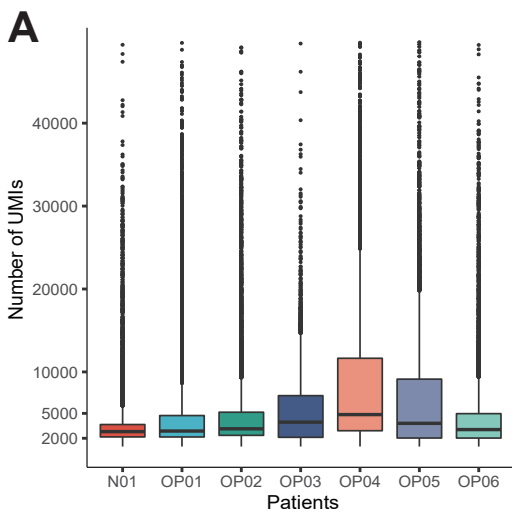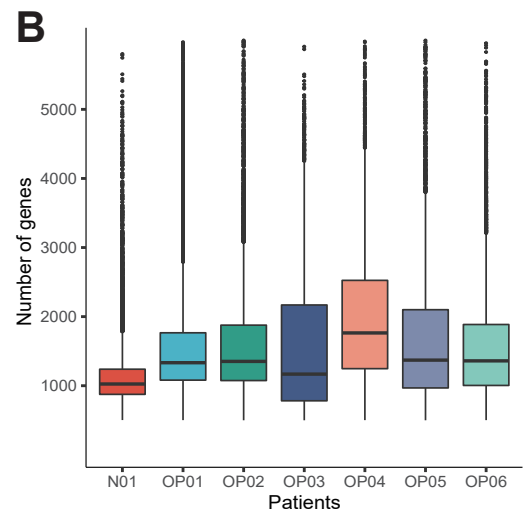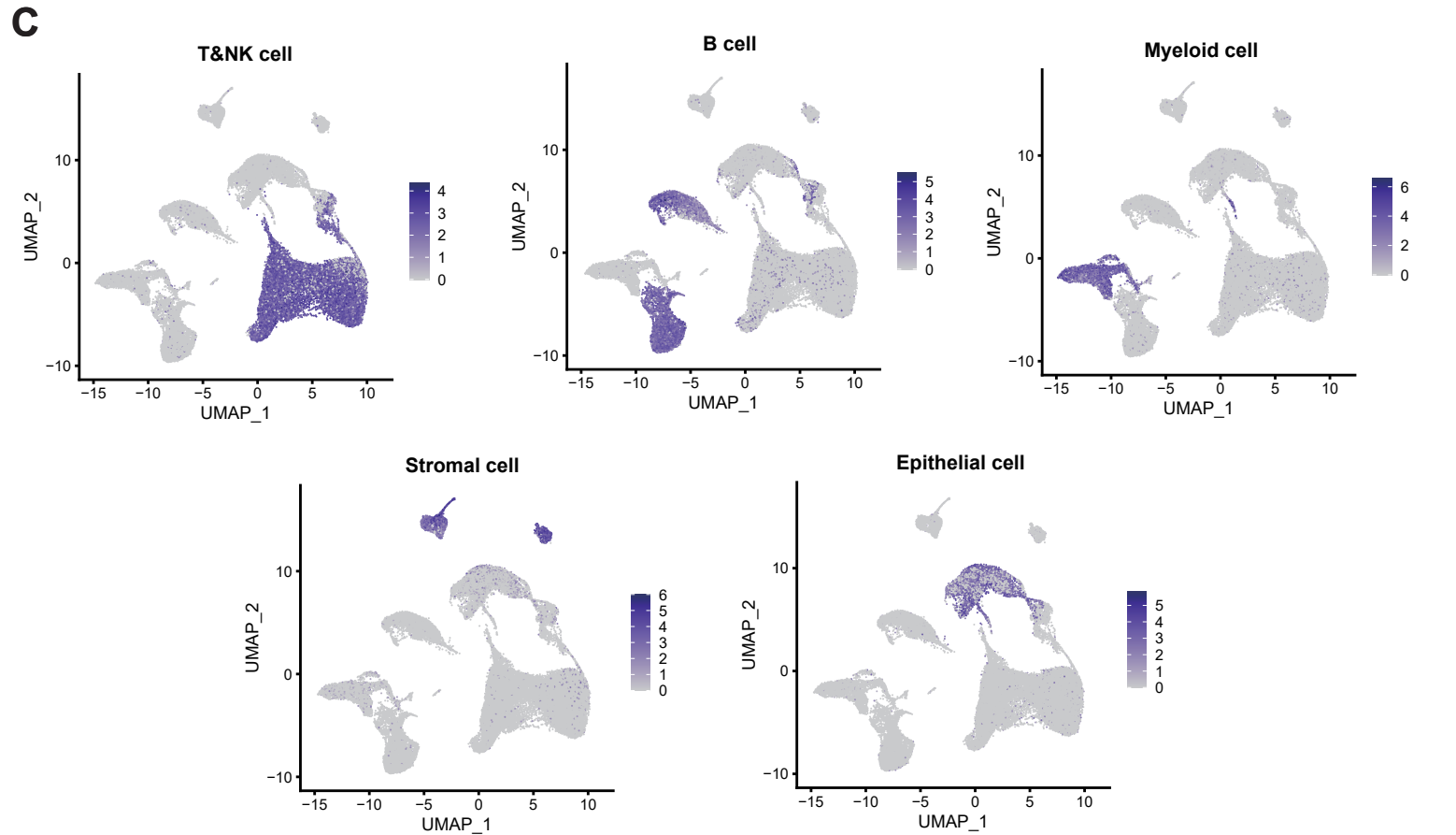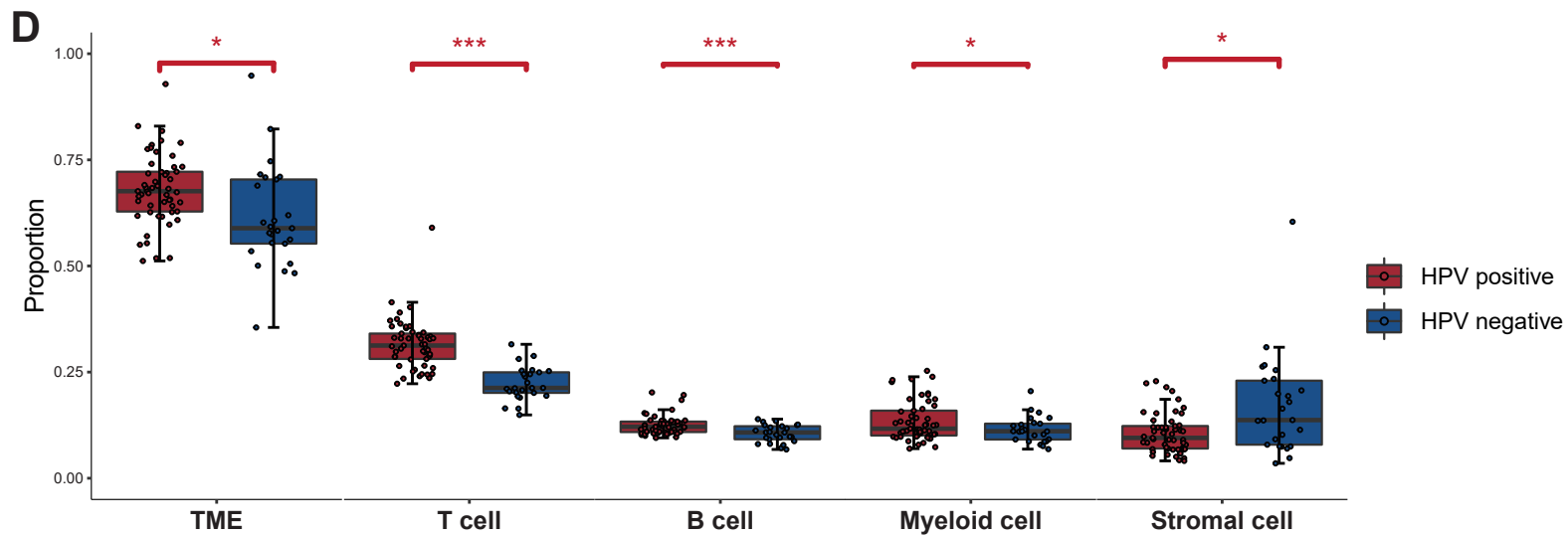

Supplement: Supplementary file 2 — Supplementary file2 (PDF 10086 kb) [file 262_2024_3789_MOESM2_ESM.pdf]

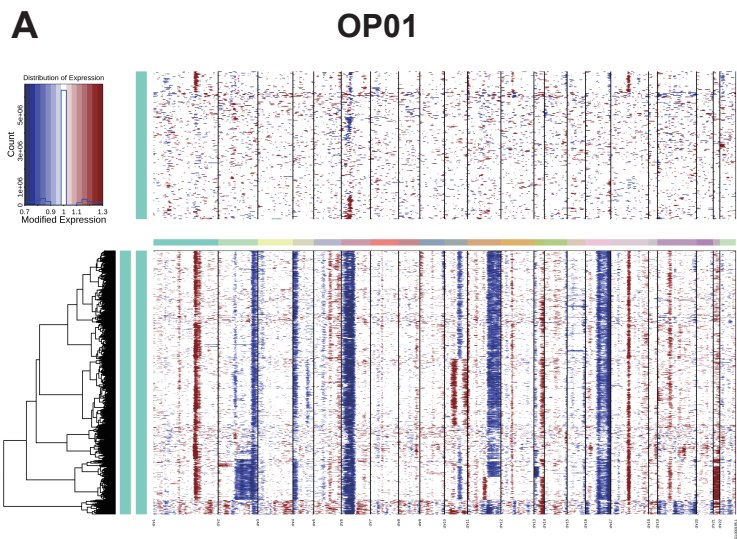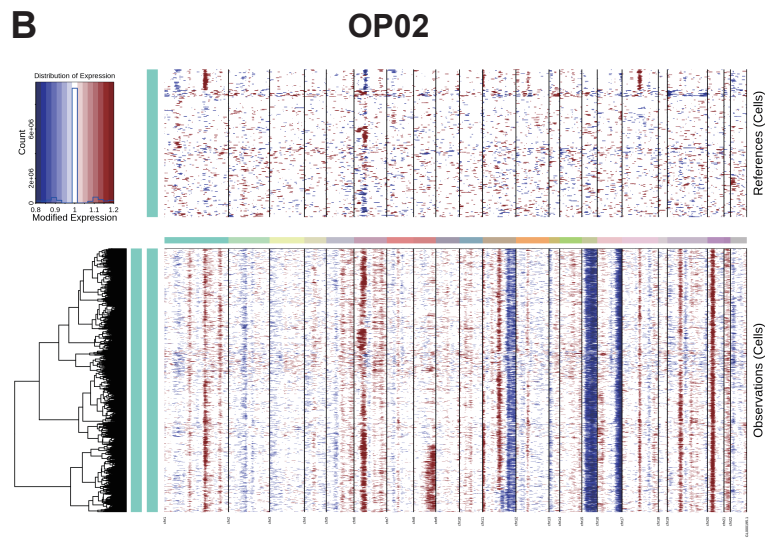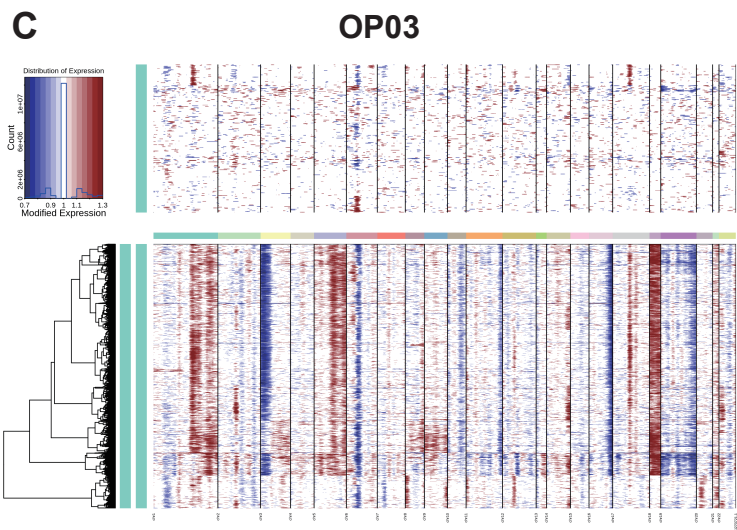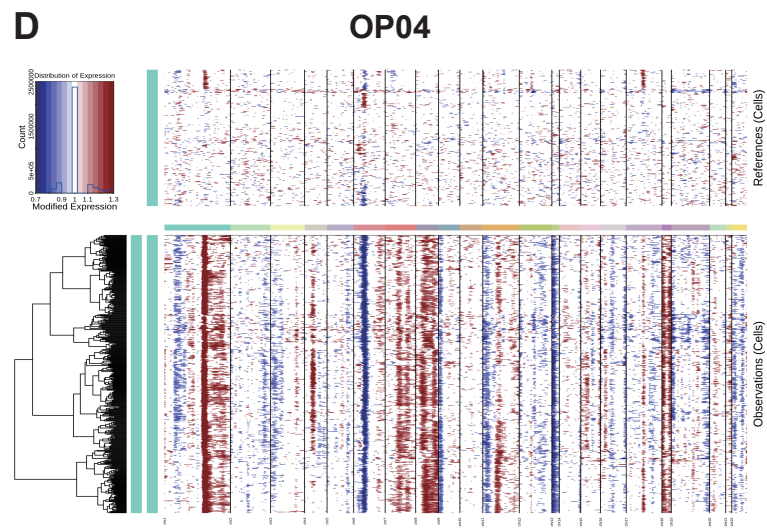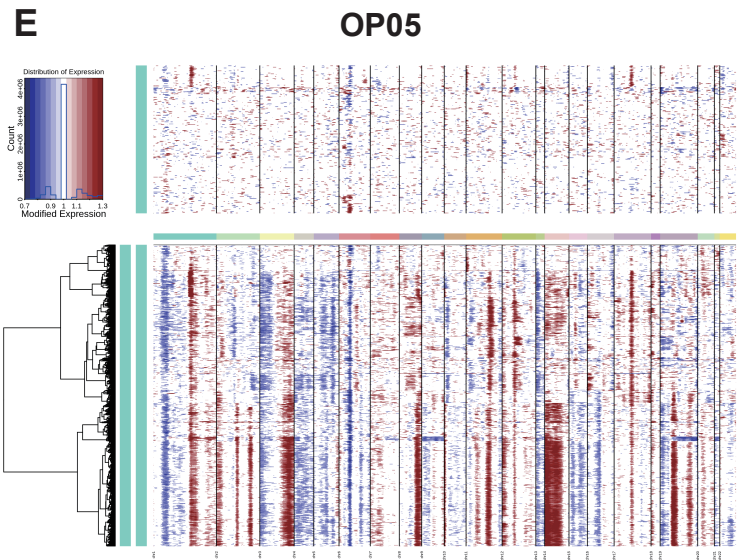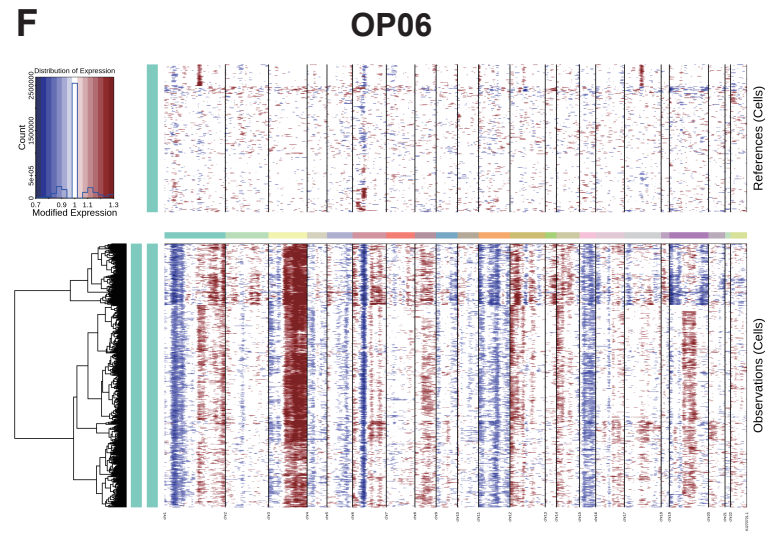

Supplement: Supplementary file 3 — Supplementary file3 (PDF 10198 kb) [file 262_2024_3789_MOESM3_ESM.pdf]

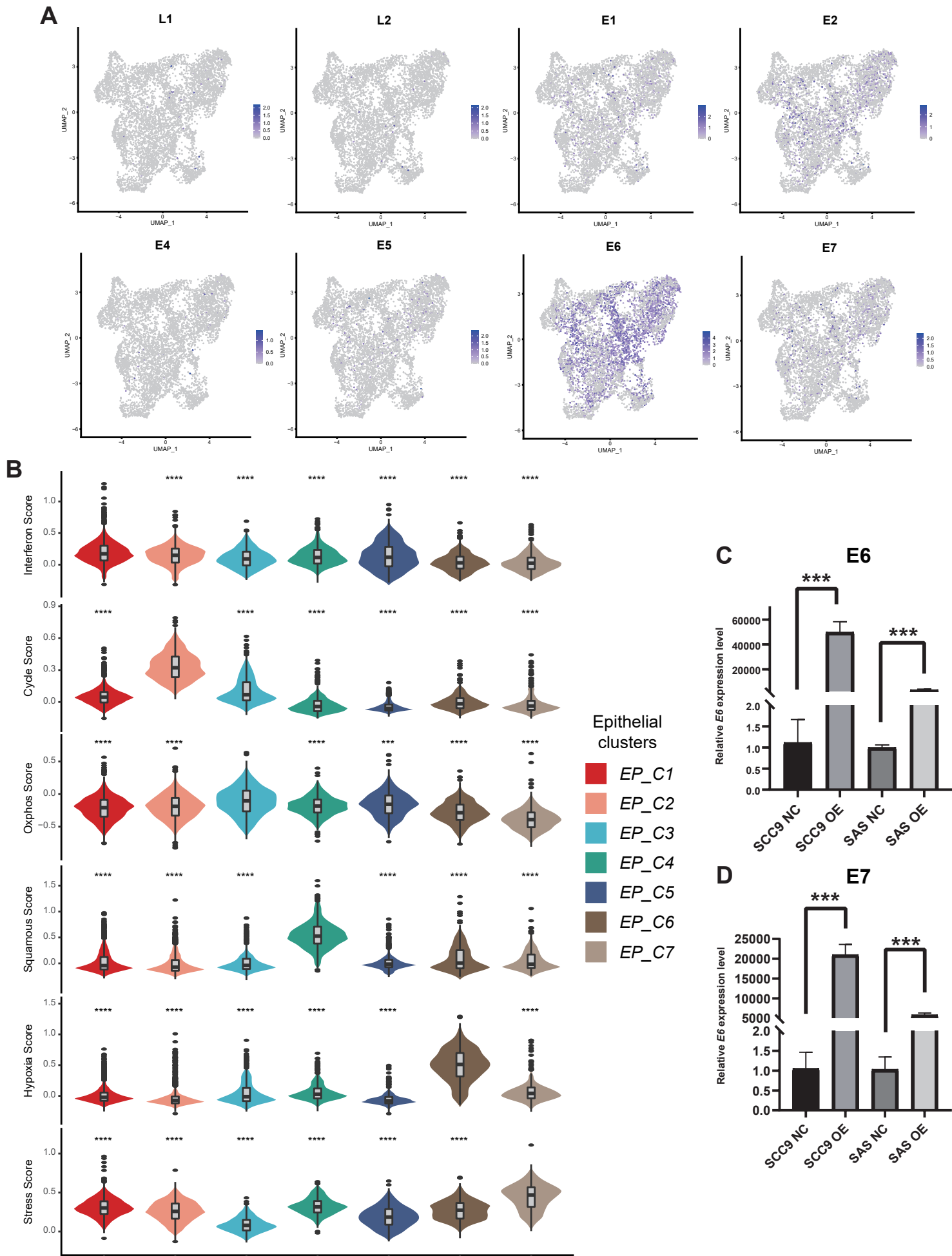

Supplement: Supplementary file 4 — Supplementary file4 (PDF 9439 kb) [file 262_2024_3789_MOESM4_ESM.pdf]

A

HPV-positive

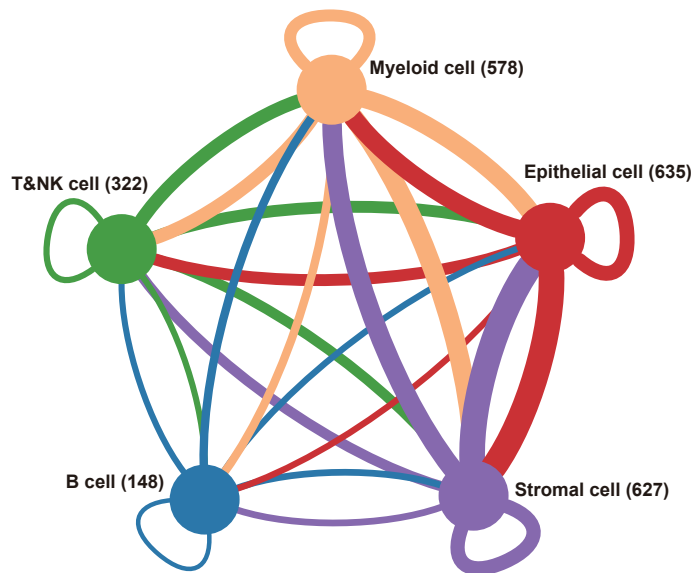

B

HPV-negative

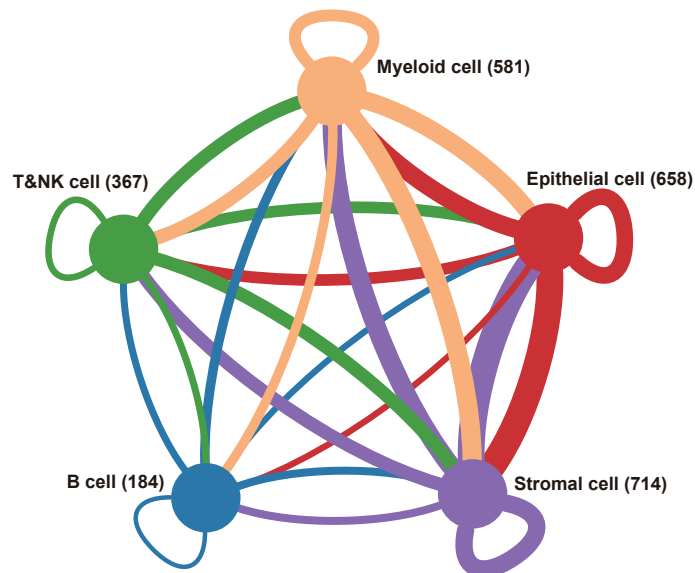

C

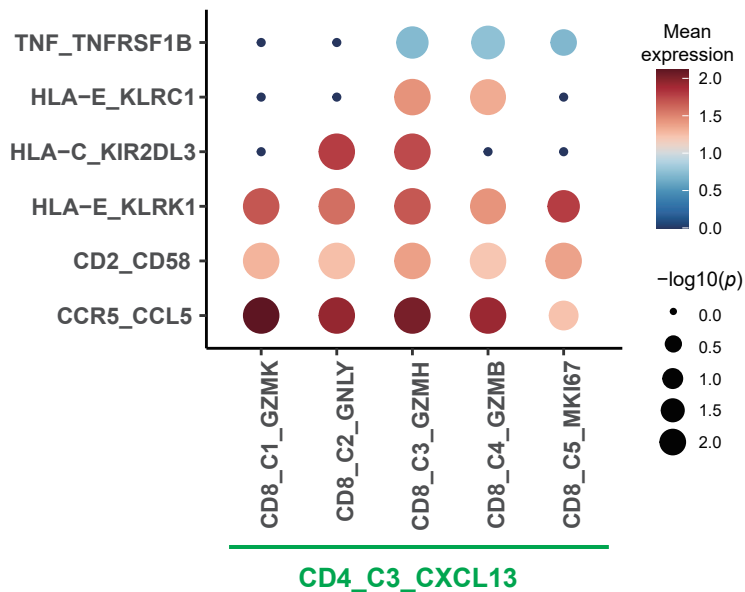

D

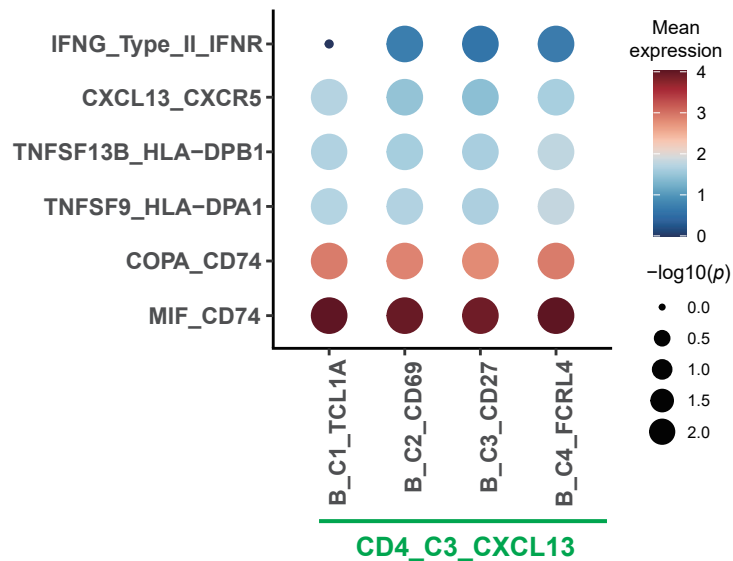

Supplement: Supplementary file 5 — Supplementary file5 (PDF 534 kb) [file 262_2024_3789_MOESM5_ESM.pdf]
